# Effects of Different Regulatory Methods on Improvement of Greenhouse Saline Soils, Tomato Quality, and Yield

**DOI:** 10.1155/2014/953675

**Published:** 2014-07-13

**Authors:** Hou Maomao, Shao Xiaohou, Zhai Yaming

**Affiliations:** College of Water Conservancy and Hydropower, Hohai University, Nanjing 210098, China

## Abstract

To identify effective regulatory methods scheduling with the compromise between the soil desalination and the improvement of tomato quality and yield, a 3-year field experiment was conducted to evaluate and compare the effect of straw mulching and soil structure conditioner and water-retaining agent on greenhouse saline soils, tomato quality, and yield. A higher salt removing rate of 80.72% in plough layer with straw mulching was obtained based on the observation of salt mass fraction in 0~20 cm soil layer before and after the experiment. Salts were also found to move gradually to the deeper soil layer with time. Straw mulching enhanced the content of soil organic matter significantly and was conductive to reserve soil available N, P, and K, while available P and K in soils of plough layer with soil structure conditioner decreased obviously; thus a greater usage of P fertilizer and K fertilizer was needed when applying soil structure conditioner. Considering the evaluation indexes including tomato quality, yield, and desalination effects of different regulatory methods, straw mulching was recommended as the main regulatory method to improve greenhouse saline soils in south China. Soil structure conditioner was the suboptimal method, which could be applied in concert with straw mulching.

## 1. Introduction

Soil salinization is one of the most serious environmental problems and is increasing steadily in many areas of the world, particularly in arid and semiarid regions [1]. Saline soils accounted for 7% of the earth's land face and increased salinization of arable land will cause 50% land loss by the middle of 21st century [2]. Based on the statistics concluded by Sheng et al. in 2008 [3], more than 1.5 billion hectares of cultivated lands in the world, approximately 77 million hectares are affected by excess salt.

Greenhouse vegetable cultivation is developing rapidly in China and accounts for 11.6% of the national agricultural acreage [4]. In recent years, tomato has quickly become one of the major vegetables grown in solar greenhouse in China because of its high potential yield, water productivity, and profitability [5]. However, every year economic loss occurs because of the reductions in greenhouse tomato productivity caused by various environmental stresses, and salt stress is one distinct type of stress for plants. Increasing salinity affected various aspects of tomato fruit, such as fruit size, blossom-end rot incidence, and fruit quality [6]. Meanwhile, salinity stress restrains the crop root-water-uptake (RWU) and influences crop growth [7]. Hu et al. [8] suggested that soil salt stress is the main factor that affects tomato yields, which can inhibit crop growth and development, thus reducing agricultural production. Furthermore, the salt stress can result in plant death in severe conditions. Besides, for tomato plant, salinity is the major factor to enhance Na^+^ uptake [9, 10], inhibit K^+^, Ca^2+^, and NO^3−^ uptake [11, 12], damage cells [13], induce oxidative stress [2], and inhibit the activity of the key enzymes and photosynthesis [14, 15].

Efforts have been made to control salinity by various technological means including drip irrigation [16], subsurface drainage [17, 18], straw mulching [19, 20], and soil conditioner application [21, 22]. Pang et al. [20] reported from a 3-year experiment that straw mulching affects the salt content in 0~40 cm soil layer, and correlation had been detected between salt content and straw mulching rates; Huang et al. [23] observed that the straw mulching prevented salt accumulation and led to a relatively constant salt level in the 0~10 cm depth, and they also observed that salts in 10~30 cm soil layer were reduced, with relatively smaller transient changes compared to those of the overlying layers when soils were mulched with straw. Other studies also reported the significant role of straw mulching in increasing crop yield [24, 25], improving crop quality [26–28], decreasing soil surface evaporation [29], and controlling soil erosion [25]. Unlike straw mulching, soil structure conditioner enhances the ability of leaching salts for soils by improving soil structure and its physical and chemical properties, thus decreasing soil salinity, and Liu et al. [30] reported that soil structure conditioner not only decreased soil salinity in plough layer by 23.08% after one season wheat cultivation, but also significantly increased soil organic matter (by about 8.02%) and crop yield (by about 10.18%).

Until now, although some studies have reported the application of regulatory methods for saline soils, the comparisons and evaluations on comprehensive effects of different regulatory methods are still lacking, and there are rare long-term and orientation studies focusing on the effects of different regulatory methods on salt dynamic changes of soils in plough layer, salt distribution in profile, soil physical and chemical properties, crop quality, and yield. In this study, a 3-year field experiment is conducted aiming at above research paucity to explore the effects of different regulatory methods on greenhouse saline soils, tomato quality, and yield; the study results are expected to provide beneficial references and practical technical supports for the improvement of greenhouse saline soils.

## 2. Materials and Methods

### 2.1. Experiment Site

The experiments were carried out at a plastic sheet covered greenhouse in Vegetables and Flowers Institute of Jiangning (latitude 31°43′ N, longitude 118°46′ E), Nanjing, China. The average annual rainfall of the experimental fields is 1106.5 mm, with the rainy season from the end of June to the middle of July. Annual temperature is 15.7°C and the average humidity is 81%. The experiments began from May 10, 2010, and lasted for about 3 years until September 25, 2013. Physical and chemical properties of original soils tested before the experiments were recorded as pH 7.91, organic matter 8.92 g kg^−1^, total N 0.85 g kg^−1^, available P 13.13 mg kg^−1^, available K 78.42 mg kg^−1^, EC 4.20 dS m^−1^, and total salt content values in soils of 0~20 cm, 20~40 cm, 40~60 cm, and 60~80 cm layer were 3.32 g kg^−1^, 1.15 g kg^−1^, 1.07 g kg^−1^, and 1.28 g kg^−1^, respectively.

### 2.2. Experimental Design

The experiments were arranged at the east side of the Vegetables and Flowers Institute; the main crop in this region was tomato. Due to several years of continuous cropping, the soils in the experimental fields had shown the moderate secondary salinification. Three regulatory methods with different additional materials were applied including rice straw mulching, soil structure conditioner, and water-retaining agent, and a control treatment with no additional materials was also set up for comparisons. The straw materials adopted 4~8 cm straw segments of paddy rice with the mulching amount of 6000 kg hm^−2^, covering the surface of soils at 20 days after the tomato seedlings were transplanted; soil structure conditioner adopted the “*Kang Di Bao*” brand developed by China Agricultural University, which could rapidly complex with salt ions in soils with its biomacromolecules, effectively decrease soil alkali and salt, and improve soil aggregate structure. The usage amount was 22.5 kg hm^−2^, sprinkling on the surface of soils according to the dilution rate of 1/1500 before the transplantation of tomato seedlings, and the conditioner would be evenly distributed in the soils with the increasing of irrigation times; water-retaining agent adopted* MP3005KM* imported from France SNF company, which was polyacrylic acid products, white powder, nontoxic noncorrosive effect, turning into irregular gel particles after absorbing water, and it possessed characters of good water absorption and retention, usage amount of which was 30 kg hm^−2^; before transplanting the tomato seedlings, the water-retaining agent was fully wetted with waters and then blended evenly with the surface soils.

One season tomatoes were cultivated from the middle of June every year during the experimental periods, and the cultivation method and process of the 3 years were similar. At the middle of June every year when all experimental conditions were implemented, the six-week-old tomato seedlings (“*Red Crown*”) were transplanted to the fields; conventional field managements were carried out fairly among the treatments; no additional light, heat, or CO_2_ was provided. Several soil ridges were made to provide a suitable growing condition for tomatoes; the ridge was 60 cm wide, 100 cm apart, and about 6 cm above the bare soil. Two line tomato seedlings were transplanted in one ridge with a 40 cm distance between them. 12 tomato seedlings were planted in a 220 cm × 60 cm block, and every 10 connected blocks were gathered as one treatment; plastic films with 80 cm depth were installed between different treatments. The experimental fields were fertilized with 700 kg hm^−2^ compound fertilizer (N : P_2_O_5_ : K_2_O = 1 : 2 : 2). Water supply was also conducted during the tomato growth period; detailed irrigation amount and its distribution were shown in Table 1. Total irrigation amount during 2011, 2012, and 2013 experimental period was 445.11 mm, 549.47 mm, and 545.86 mm, respectively. Frequency irrigation method with small waters was applied in 2011, and traditional one as local habits was conducted in both 2012 and 2013.

### 2.3. Sampling and Measurements

Soil salinity: 4 salt sensors were buried fixedly into 10 cm depth of topsoil to detect the electrical conductivity dynamic changes of soils in plough layer with different regulatory methods. On the other hand, the mass fraction of salt in soil profile (0~20 cm, 20~40 cm, 40~60 cm, 60~80 cm) was also determined before transplanting and after the harvest of tomatoes.

Tomato quality: at tomato maturity, 2 marketable tomato fruits were harvested from one plant, and about 10 g tomato flesh per fruit was taken along the longitudinal axis (20 fruits were randomly chosen in one treatment) and then homogenized for the quality measurements. Vitamin C content was measured by the 2,6-dichloroindophenol titrimetric method; soluble sugar was measured by the anthrone method; soluble protein was measured by the Coomassie brilliant blue method; nitrate content was measured by the ultraviolet spectrophotometry method [31, 32].

Weight of individual fruit: for each treatment, 30 tomato fruits with a red or orange color were collected randomly to determine the weight of tomato fruit. Tomato weight (for each treatment) was the average from 30 individual fruits weight.

### 2.4. Data Analysis

The differences among treatments were analyzed by Duncan's new multiple range test [33].

Principal component analysis method combined with entropy weight coefficient model was applied to evaluate the effects of different regulatory methods, and the main evaluation indexes included the comprehensive quality of tomato fruit, tomato yield, and desalination rate.

### 2.5. Evaluation of Regulatory Methods

Entropy weight coefficient model was a method dealing with multidimensional data [34, 35]. In this study, the model was established based on the data in Table 4. And the modeling approach was shown below [36].

Supposing that there are *n* evaluation indexes and *m* regulatory methods, *m* regulatory methods corresponding with *n* indexes obtain the following matrix:

(1) $$\begin{array}{c} R={\left(r_{ij}\right)}_{m\times n}, \end{array}$$

where *r*_*ij*_ is the *j*th evaluation index of the *i*th regulatory method. To *r*_*j*_, there is information entropy (average amount of information after excluding redundancy) as follows:

(2) $$\begin{array}{c} E_{j}=-\overset{m}{\underset{i=1}{\sum }}p_{ij}\ln p_{ij},\;\left(j=1,2,3,\ldots ,n\right). \end{array}$$

And *p*_*ij*_ are calculated from the following formula:

(3) $$\begin{array}{c} p_{ij}=\frac{r_{ij}}{\sum_{i-1}^{m}r_{ij}}. \end{array}$$

The entropy value of *j*th index is

(4) $$\begin{array}{c} e_{j}=\frac{1}{\ln m}E_{j},\;\left(j=1,2,3,\ldots ,n\right). \end{array}$$

The objective weight of *j*th index is

(5) $$\begin{array}{c} \theta_{j}=\frac{\left(1-e_{j}\right)}{\sum_{i=1}^{n}\left(1-e_{j}\right)},\;\left(j=1,2,3,\ldots ,n\right). \end{array}$$

It is clear that

(6) $$\begin{array}{c} 0\leq \theta_{j}\leq 1;\;\overset{n}{\underset{j=1}{\sum }}\theta_{j}=1. \end{array}$$

This study takes the subjective information into the calculations; the comprehensive weight can be obtained by combining the subjective weight *w*_1_, *w*_2_, *w*_3_,…, *w*_*n*_ of decision makers with the objective weight *θ*_*j*_ (*j* = 1,2, 3,…, *n*) as follows:

(7) $$\begin{array}{c} \alpha_{j}=\frac{\theta_{j}\bar{\omega_{j}}}{\sum_{j=1}^{n}\theta_{j}\bar{\omega_{j}}},\;\left(j=1,2,3,\ldots ,n\right). \end{array}$$

Optimal index value of each column is recorded as *r*_j_*, indexes can be divided into 2 different parts, profitability index and damnous index. For profitability index, a higher value is better; for damnous index, a lower value is better. The calculation method is listed as follows:

(8) $$\begin{array}{c} d_{ij}=\{\begin{array}{cc} \frac{r_{ij}}{r_{j}^{*}}, & r_{j}^{*}=\max\left\{r_{ij}\right\}\\ \frac{r_{j}^{*}}{r_{ij}}, & r_{j}^{*}=\min\left\{r_{ij}\right\}. \end{array} \end{array}$$

Entropy coefficient value (better regulatory method will obtain higher entropy coefficient evaluation value) of each regulatory method can be calculated from

(9) $$\begin{array}{c} \lambda_{i}=\overset{n}{\underset{j=1}{\sum }}\alpha d_{ij},\;i=1,2,3,\ldots ,m. \end{array}$$

## 3. Results and Discussion

### 3.1. Changes of Soil Electrical Conductivity (EC)

**Figure 1:**
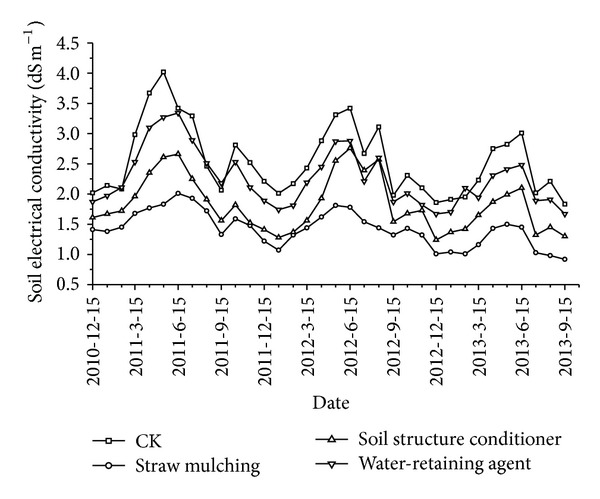
Soil electrical conductivity in 10 cm depth of plough layer with different regulatory methods.

Figure 1 showed the changes of soil electrical conductivity monitored in permanent position with different regulatory methods. In all 3 seasons, changes of soil EC in plough layer were not obvious from October to the following February; this was probably because there were less evaporations after entering the winter, the vertical migration of soil saline was mild in this period, soil EC in plough layer of CK was significantly higher than that of other treatments, and water-retaining agent had some effects on decreasing soil EC in plough layer, while the effects were not evident as straw mulching and soil structure conditioner. From March every year, EC showed obvious rise trend; this may relate to the dry and windy climate and stronger evaporation effects in spring [20]. June to September was the growth period of tomatoes, the evaporations in this period were especially intense, and this aggravated the salt accumulation in soils of plough layer; after irrigation, soil EC in plough layer showed different decreases, but which rose again soon with time, fluctuating wildly; in this stage, soil EC in plough layer with straw mulching treatment was found to change most mildly. This was mainly because the straw mulching lowered the water loss and increased the water storage in surface soils, slowing down the salt accumulation in plough layer [30, 37]. Soil structure conditioner also had significant effects on decreasing soil EC, and it could be because the 3-year application of soil structure conditioner improved the permeability of soils in plough layer, accelerated the elution of soil salts, and lessened the salt accumulation [38]. Wu et al. [39] observed a 41.8% increase of soil permeability with macromolecule soil structure conditioner. The 3-year locating observation in this study showed that the 3 regulatory methods could all decrease the soil EC in plough layer; decreasing effects from high to low were straw mulching > soil structure conditioner > water-retaining agent. Straw mulching decreased surface soil EC by 44.57% over the 3 years; the result was reported higher by Badía [40] that straw mulching decreases soil EC by 2.5 times in semiarid areas.

### 3.2. Changes of Soil Salt in Plough Layer

**Figure 2:**
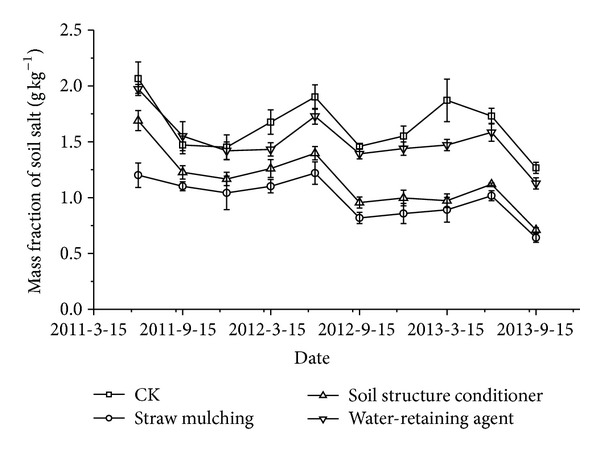
Mass fraction of soil salt in plough layer with regulatory methods.

Figure 2 showed the changes of soil salt in plough layer varying with time. In the 3 seasons, soil salt content of plough layer in June was higher than that in September; this is mainly because there was almost no water supply before June, and the evaporation effects were considerably strong; while September was the maturity month for tomatoes, after the irrigations of one season tomato, soil salts in plough layer decreased. In this respect, our result was similar to Guo's research; he suggested that when the irrigation quota was >2700 m^3^ hm^−2^, irrigation was helpful to the leaching of salt in the 0~100 cm soil profile, while when the irrigation quota was <1800 m^3^ hm^−2^, irrigation accelerated the salt accumulation in surface soil. In June 2011, the soil salt content in plough layer of CK, straw mulching, soil structure conditioner, and water-retaining agent treatment decreased 62.35%, 36.14%, 50.90%, and 59.64%, respectively, compared to that of the original soils before experiment. From June 2010 to September 2013, soil salt content in plough layer presented a fluctuant reduction, which reached the lowest value detected in September 2013, and the soil salt content values of straw mulching, soil structure conditioner, and water-retaining agent treatment at the moment were 49.48%, 43.91%, and 11.02% lower than that of CK. Bezborodov et al. [41] observed a 20% increase in surface soil salinity of the nonmulching treatments compared to the mulching treatments; it was reported higher in our study which may result in the differences of soil evaporation caused by experimental condition. Among 3 regulatory methods, the desalination effects of straw mulching were especially satisfactory. Desalination rate of soil salts in plough layer reached a high value of 80.72% after 3 years' experiments; this may be due to the effects of evaporation suppression, and previous studies have shown that use of mulches significantly reduced soil evaporation [20, 42].

### 3.3. Profile Distribution of Soil Salt with Different Regulatory Methods

**Figure 3:**
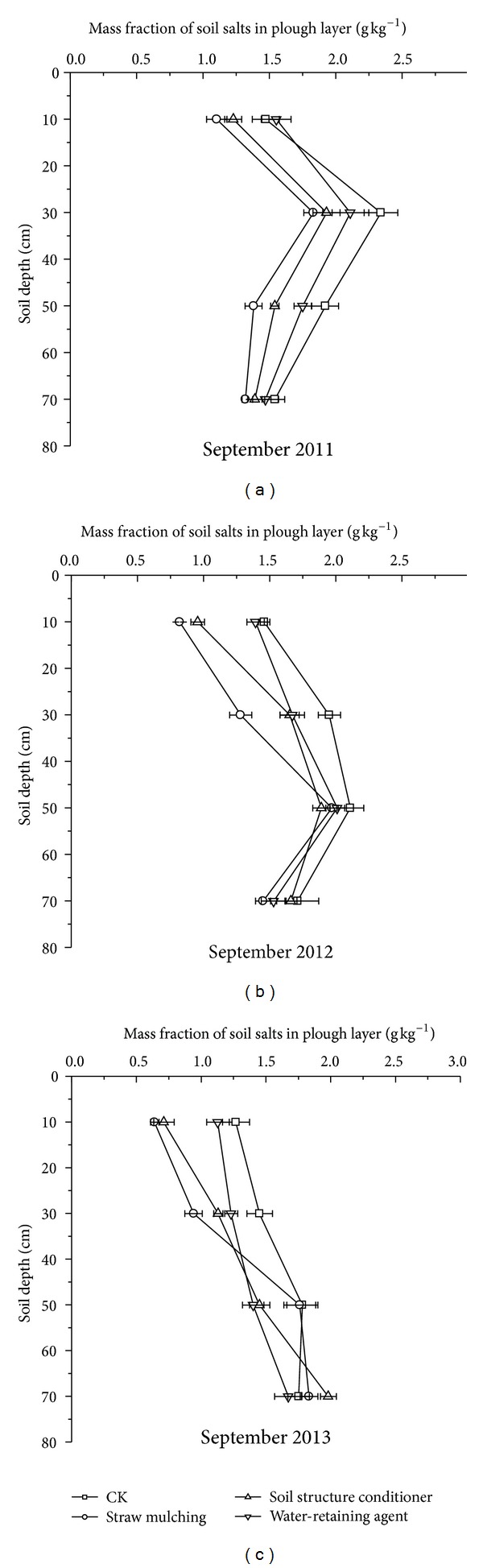
Profile distribution of soil salts in different experimental seasons.

Figure 3 showed the profile distribution of salt soils with different regulatory methods during the experimental periods. Characters of profile distribution of salt soils had great differences among different experimental stages. Before the experiment, most salts accumulated in the surface soil; salt content in 0~20 cm soil layer accounted about 50% for the total salt content. After one year experiment, salts in surface soil decreased obviously, which were kept in the range of 1.0 g kg^−1^~1.6 g kg^−1^; however, salt content of 20~40 cm soil layer increased dramatically, of which 40~60 cm soil layer also showed the rising trend. Zhao et al. [43] reported that topsoil salinity with 3 years' straw mulching decreased by 4.5~31.6% but mulch treatments moderately increased soil salinity in subsoil (20~40 cm) layer. After two years' experiment, soil salts moved to the deeper soil layer; salt content in 40~60 cm soil layer was the highest at the moment. In 2013 season, salt content in surface soils had fallen to the lowest, and high soil salinity appeared at the 40~60 cm and 60~80 cm soil layer.

Salts in surface soil with straw mulching decreased most dramatically in all 3 experimental seasons; a similar study also reported that straw mulching affected vertical distribution of salts within 0~100 cm soil depth [20]. In September 2013, salt content in 60~80 cm soil layer reached the highest value, and of which CK, straw mulching, soil structure conditioner, and water-retaining agent were 36.72%, 42.97%, 54.69% and 30.47% higher than that of original soils tested in May, 2010. Among the 3 regulatory methods, salt content of 60~80 cm layer with soil structure conditioner was the highest; this could be explained by the better permeability of soils with soil structure conditioner treatment [44], which promoted the soil salts to move with irrigation waters from surface soil layer to deeper soil layer.

### 3.4. Soil Organic Matter in Plough Layer

**Figure 4:**
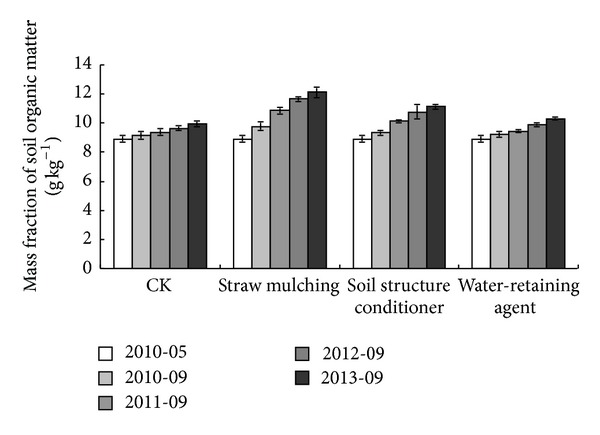
Changes of organic matter content in plough layer with different regulatory methods.

Soil organic matter content was one of the most important indexes to evaluate the soil improvement result.  Figure 4 showed the changes of organic matter content in plough layer with different regulatory methods during experimental periods. In general, the soil organic matter content in plough layer presented a rising trend with different regulatory methods. The increment of soil organic matter content with straw mulching increased most significantly; there were maybe 2 reasons, one because the rice straw contained lots of organic ingredients [45, 46], or because the straw mulching reduced the runoffs and so lowered the nutrient loss [47]. Feng et al.'s [48] research also showed that straw mulching proved to be very advantageous in improving soil subsurface water content and conserving nutrients. Organic matter content in plough soil layer with soil structure conditioner treatment increased by 24.66% compared to the original soils. Similar result could be found in Xu et al.'s [49] research that organic matters contents in the soil conditioner treated orchard soils were all significantly (*P* < 0.05) higher than those in the nontreated soil.

This mainly because that the biomacromolecules of soil structure conditioner improved soil aggregate structure and basic character, thus help conserved the organic matter of soils. However, water-retaining agent had smaller effect on increasing the soil organic matter content in plough layer.

### 3.5. Changes of Soil Nutrient in Plough Layer

Tomato is a very exigent plant for nutrients.  Table 2 showed the changes of soil nutrient in plough layer with different regulatory methods (measured in September each year). Available N increased with time after being treated with different regulatory methods; the increment of straw mulching was maximum reaching 44.74% observed in 2013. This was in agreement with Tu et al.'s [28] early study which concluded that straw mulching further enhanced available N content by 30% over 2 years, and they also noticed a 182~285% increase in potential mineralizable N. Different from the change laws of available N, available P presented a decline trend with time; among the 3 regulatory methods, available P with soil structure conditioner treatment decreased most evidently, and this indicated that a greater usage of P fertilizer was needed when applying soil structure conditioner; straw mulching reserved more available P compared to other methods, the available P content of which was 27.32%, 20.63%, and 8.48% higher than that of CK in 2011, 2012, and 2013, respectively; however, available P content with water-retaining agent treatment changed little and was only 3.49% higher than that of CK in 2013. Available K content of straw mulching treatment presented a significant improvement, recording as 107.9 mg kg^−1^ in 2013; on the contrary, soil structure conditioner treatment decreased by 21.44% compared with that of the original soils before experiment, and the dramatic decrease appeared at 2012~2013 period. Therefore, soil structure conditioner was suggested to be unfavorable in conserving soil P and K. In the relationship between soil structure conditioner application and soil P content changes, our study result was in agreement with Liu et al.'s [30] study but differed from Liu et al.'s [50]. Previous studies have shown that soil P was easy to lose [51, 52], and soil conditioner could improve soil permeability [39, 53] thus easier to cause P leaching. From a pure point of soil nutrient conservation, straw mulching proved to be a better regulatory method.

### 3.6. Principal Component Analysis of Tomato Quality Indexes

For evaluating the effects of different regulatory methods on tomato comprehensive quality, the principal component of tomato quality indexes needed to be extracted. Main quality indexes of tomato fruit during the experimental seasons were shown in Table 3. Taking 2011 as an example, principal component of the quality indexes in Table 3 was extracted following the principle of “eigenvalue >1, cumulative contribution rate >80%” [54, 55]. Using SPSS 14.0 software to calculate the principal component of the samples, the calculated eigenvalue, contribution rate (*r*_*c*_), and cumulative contribution rate (*r*_*T*_) were displayed as in Table 3. The only principal component (*f*) reflected the evolution information of fruit density (*X*_1_), fruit volume (*X*_2_), soluble solid (*X*_3_), total acid (*X*_4_), vitamin C (*X*_5_), and sugar/acid ratio (*X*_6_). Cumulative contribution rate was calculated as 85.862%, reserving vast raw information of the quality indexes. The comprehensive quality index of tomato fruit under different regulatory methods in 2011 was shown in the second column of Table 4. Regulatory method with higher value of comprehensive quality index obtained better tomato quality. Therefore, in the 2011 season, the comprehensive quality of tomato with CK treatment was optimal.

Principal component analysis of tomato quality indexes showed that comprehensive quality index of tomato fruit with CK treatment was optimal in all 3 years. This may be explained by the higher soil salinity in CK compared to other methods. Dorais et al. [56] have shown that higher salinity (<9 ms cm^−1^) improved tomato quality but negatively affected the yield. Similar results were also obtained by Takahata and Miura's [57] study that salt stress increased sugar and soluble solid concentration. Amjad et al. [58] also noted that fruit quality characteristics (total soluble solids, titratable acidity, pH, and dry matter %) were significantly improved by increasing salinity, except for fruit size. However, although many studies have shown a positive relationship with soil salinity and tomato quality, it should be noticed that high salinity caused higher incidence of tomato blossom-end rot (BER) [59–61] and reduced marketable tomato yield [6].

### 3.7. Entropy Weight Coefficient Evaluation of Different Regulatory Methods

**Figure 5:**
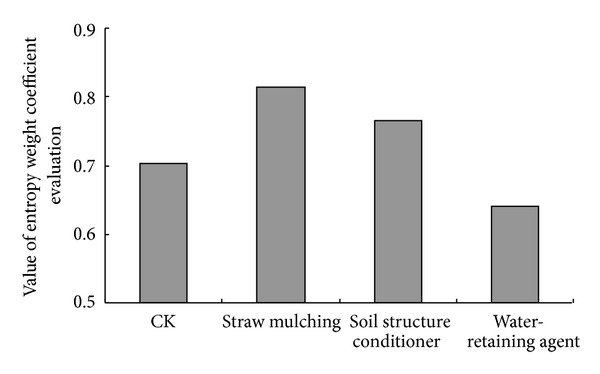
Value of entropy weight coefficient evaluation with different regulatory methods.

In this study, profitable indexes included the tomato comprehensive quality index and the tomato yield; damnous index included the salt content of soils in plough layer (Table 5). In this study, soil desalination was expected to be the most important evaluation index. Subjective weight was assigned as 0.15, 0.7, and 0.15 corresponding with comprehensive quality index, soil salt in plough layer, and tomato yield, respectively. Considering the 3 evaluation indexes in the 3 years, straw mulching proved to be the best regulatory method with highest entropy weight coefficient of 0.8149 based on the calculation result in Figure 5, followed by soil structure conditioner treatment, entropy weight coefficient of which was recorded as 0.7644. This result indicated that straw mulching was the better method scheduling with the compromise between the soil desalination and the improvement of tomato quality and yield, and soil structure conditioner was the suboptimal regulatory method. It was worth noticing that straw mulching increased tomato yield by 24.19%~29.55% in the 3 seasons. Taparauskiene and Miseckaite [62] also observed a higher increase of crop yield by 56% with straw mulching within a 2-year experiment in subhumid area.

## 4. Conclusion

Our results have shown that EC of greenhouse saline soils in plough layer presented a fluctuant reduction, of which straw mulching treatment decreased most significantly. Among the 3 regulatory methods, straw mulching had a better effect on removing soil salts in plough layer; removing rate reached 80.72% over 3 years. In addition, straw mulching significantly increased the soil organic matter content by 21.69% in plough layer compared to CK and was conductive to reserve the soil available nutrient including available N, available P, and available K. A greater usage of P fertilizer and K fertilizer was needed when applying soil structure conditioner.

Considering the evaluation indexes including tomato quality, yield, and desalination effects of different regulatory methods, straw mulching was supposed to be the best method with highest entropy weight coefficient of 0.8149 and was recommended as the main regulatory method to improve greenhouse saline soils in south of China. Soil structure conditioner was the suboptimal method, which could be applied in concert with straw mulching.

## Figures and Tables

**Table 1:** Detailed irrigation amount and its distribution (mm).

Date	2011	2012	2013
June 15	40.21	50.98	52.24
June 29	36.92	65.54	61.18
July 7	46.21		
July 16	42.28	65.33	64.33
July 24	36.62		
August 5	40.99	78.64	76.54
August 13	32.16		
August 19	48.72	72.12	74.98
August 29	41.21	79.33	74.43
September 7	40.95	69.21	72.12
September 14	38.84	68.32	70.04

Total	445.11	549.47	545.86

**Table 2:** Changes of soil nutrient in plough layer with different regulatory methods. Columns with the same letter represent values that are not significantly different at the 0.05 level of probability according to Duncan's multiple range test. Each value is the mean ± SD (*n* = 3).

Treatment	Available N (mg kg^−1^ )			Available P (mg kg^−1^)			Available K (mg kg^−1^)		
	2011	2012	2013	2011	2012	2013	2011	2012	2013
CK	53.33 ± 2.50^b^ *l* ^1^ ^1^	61.08 ± 3.26^c^	65.43 ± 4.84^c^	10.14 ± 0.39^c^	9.84 ± 0.37^bc^	8.61 ± 0.10^b^	75.13 ± 3.96^b^	72.35 ± 2.04^b^	74.32 ± 4.21^b^
Straw mulching	62.38 ± 5.56^a^	78.28 ± 3.61^a^	90.29 ± 6.53^a^	12.91 ± 0.34^a^	11.87 ± 0.24^a^	9.34 ± 0.17^a^	88.88 ± 2.27^a^	100.49 ± 8.81^a^	107.09 ± 5.58^a^
Soil structure conditioner	53.63 ± 1.53^b^	70.76 ± 1.71^b^	80.44 ± 4.09^b^	11.17 ± 0.25^b^	9.63 ± 0.50^c^	7.14 ± 0.32^c^	71.85 ± 4.60^b^	70.95 ± 3.19^b^	61.61 ± 1.57^c^
Water-retaining agent	52.34 ± 2.23^b^	65.23 ± 2.62^c^	74.14 ± 7.07^bc^	10.64 ± 0.41^bc^	10.38 ± 0.27^b^	8.97 ± 0.09^b^	77.76 ± 1.47^b^	70.70 ± 3.57^b^	77.91 ± 7.25^b^

**Table 3:** Main quality indexes of tomato fruit. Columns with the same letter represent values that are not significantly different at the 0.05 level of probability according to the Duncan's multiple range test.

Year	Treatment	Density (g cm^−3^)	Volume (cm^3^)	Soluble solid (%)	Total acid (g 100 g^−1^)	Vitamin C (mg 100 g^−1^)	Sugar/acid ratio
2011	CK	0.940^a^	114.34^b^	7.66^a^	0.672^b^	13.82^a^	9.61^a^
	Straw mulching	0.957^a^	138.98^a^	6.95^b^	0.598^c^	11.80^c^	8.79^c^
	Soil structure conditioner	0.948^a^	127.87^ab^	7.12^ab^	0.621^c^	12.54^b^	9.04^b^
	Water-retaining agent	0.948^a^	122.05^b^	7.25^ab^	0.746^a^	13.55^a^	9.11^b^

2012	CK	0.937^a^	110.48^b^	7.58^a^	0.666^ab^	14.12^a^	9.87^a^
	Straw mulching	0.949^a^	130.66^a^	7.02^b^	0.613^b^	11.89^c^	9.01^b^
	Soil structure conditioner	0.944^a^	122.32^ab^	7.28^ab^	0.627^b^	12.64^b^	9.12^b^
	Water-retaining agent	0.940^a^	128.68^a^	7.37^a^	0.708^a^	13.85^a^	9.29^b^

2013	CK	0.939^a^	122.48^a^	8.07^a^	0.622^b^	14.11^a^	10.04^a^
	Straw mulching	0.948^a^	124.93^a^	7.14^b^	0.646^ab^	12.54^b^	9.37^b^
	Soil structure conditioner	0.941^a^	125.28^a^	7.11^b^	0.699^a^	12.19^b^	9.25^c^
	Water-retaining agent	0.945^a^	121.15^a^	7.08^b^	0.638^ab^	14.09^a^	9.41^b^

**Table 4:** Weight coefficient and contribution rate of main ingredients.

		*X*_1_	*X*_2_	*X*_3_	*X*_4_	*X*_5_	*X*_6_	Eigenvalue	*r*_*c*_ (%)	*r*_*T*_ (%)
2011	*f*_1_	−0.961	−0.997	0.962	−0.670	0.977	0.953	5.152	85.862	85.862

2012	*f*_1_	−0.690	−0.997	0.792	−0.070	0.573	0.886	3.215	53.582	53.582
	*f*_2_	−0.720	−0.051	0.596	−0.997	0.820	0.402	2.703	45.054	98.636

2013	*f*_1_	0.096	−0.912	0.369	0.916	0.932	0.555	2.995	49.911	49.911
	*f*_2_	−0.910	−0.054	0.899	0.172	0.283	0.801	2.389	39.820	89.731

**Table 5:** Comprehensive quality index, soil salt content in plough layer, and tomato yield.

Treatment	Comprehensive quality index			Salt in plough layer (g kg^−1^)			Tomato yield (t hm^−2^)		
	2011	2012	2013	2011	2012	2013	2011	2012	2013
CK	3.014	2.872	2.617	1.47	1.46	1.27	103.84	108.68	112.47
Straw mulching	1.000	1.000	1.713	1.10	0.82	0.64	136.56	145.73	157.22
Soil structure conditioner	1.757	1.554	1.000	1.23	0.96	0.71	122.39	139.28	134.55
Water-retaining agent	2.339	1.830	1.956	1.55	1.39	1.13	128.96	135.47	145.71
